# Two-Step Glass Molding Process for Forming Glass Edges with Obtuse Angles for Mobile Displays

**DOI:** 10.3390/mi13071032

**Published:** 2022-06-29

**Authors:** Jeongyeon Park, Sungho Chang, Dongwon Lee, Hyeonhwa Lee, Bongchul Kang, Jongsu Kim

**Affiliations:** 1 Molding & Metal Forming R&D Department, Korea Institute of Industrial Technology, Bucheon 14441, Korea; parkjy@kitech.re.kr (J.P.); ldw@kitech.re.kr (D.L.); vikar02@kitech.re.kr (H.L.); 2 Gaon-Solutec Co., Incheon 21694, Korea; gaon2013@gosolutec.com; 3 School of Mechanical Engineering, Inha University, Incheon 22212, Korea; 4 School of Mechanical Engineering, Kookmin University, Seoul 02707, Korea

**Keywords:** full three-dimensional cover glass, glass molding press, mold/punch design, two-step compression process

## Abstract

The domain of edge displays with 2.5D or 3D curved designs has been expanded to improve user convenience. The currently available 3D cover glass offers a limited curvature radius of at least 5 mm and a curvature less than 88°, due to limitations in the undercuts and formability of parts. The development of a full 3D cover, applicable to next-generation displays, requires cover glass molding technology with a curvature exceeding 90°. Here, a mold design and molding process, which addresses the current limitations by dividing the existing glass molding press (GMP) process into two stages, is proposed. The bending geometry of the glass prepared on the basis of the proposed mold design plan during single-step compression forming and two-step compression forming was predicted using commercial analysis software. A molding product with a curvature radius of 2.5 mm and an angle of curvature of 138.9° was produced when process conditions with bending by up to 180° with no damage were applied during actual forming experiments. Further research on annealing and cooling processes of GMP is expected to enable the design and process implementation to manufacture curved glass with a single curvature of at least 90° and multiple curvatures.

## 1. Introduction

Performance enhancements of smart devices have been sought on a wide scale to meet the market demands arising from commercial competition. To this end, efforts have been devoted toward differentiation by concentrating technologies to achieve a user-friendly design. A curved display design has widely been applied because of its ergonomic capabilities and improved user convenience due to expansion of the displayed domain [1,2]. A number of smartphone manufacturers have launched devices with curved edge displays or curvature on the rear edge [3,4]. The implementation of such curved edge displays essentially requires curvature molding technology for the cover glass along with the display technology. Cover glass designs are mainly classified into 2D, 2.5D, and 3D, according to the curvature application pattern [5]. Two-dimensional cover glass is a commonly used flat-plate-type cover glass. It can be mass-produced at a low cost because it can be manufactured through simple machining; however, the disadvantage of 2D cover glass is that it cannot be used to manufacture ultrathin smart devices with the desired aesthetics and functionality [6]. 2.5D cover glass is an intermediate stage of development from 2D to 3D cover glass. In this case, the part where the edge of the front part meets that of the side part is further processed such that its shape resembles a 3D pattern. This cover glass, however, has shortcomings similar to those of the 2D cover glass, although it is prepared through the same process as that of the 2D cover glass and has the benefit of mass production. Three-dimensional cover glass is produced by molding flat cover glass to achieve a freeform surface geometry (3D) through hot forming. It has the benefit of allowing ultrathin smart devices with high functionality to be manufactured, despite its smaller-scale production and higher cost compared with the conventional 2D and 2.5D cover glasses. Thus far, only a few technologies [7,8,9,10,11] and design patents [12,13,14,15] have been implemented to achieve the desired specifications in commercial 3D cover glasses, namely, a curvature radius not exceeding 5 mm and a curvature of less than 88°. However, limited technical research has been conducted on the actual manufacturing process of such glasses. The application of curved displays to the edges of mobile devices by overcoming the above-mentioned technical limitations would enable the production cost to be lowered by eliminating metal buttons on the sides and meeting the latest technical requirements that prioritize waterproof and dustproof functions [16,17]. Furthermore, as the metal body frame is inserted inside the edge glass, communication failure that may intermittently occur depending on the user’s grip can be fundamentally overcome. To meet these design and functional requirements, it is necessary to develop new types of displays with an edge curvature of 90–180°.

In general, the glass molding press (GMP) process that performs molding by applying heat and pressure to glass is used as a method for manufacturing curved displays [18,19]. In the GMP process, glass is heated to the transition temperature and pressed using a mold to achieve the desired geometry. The final geometry can be achieved with no additional processing because the geometry provided to the glass is replicated through the mold geometry, and the production efficiency is significantly higher than that of a typical material removal process. However, despite several years of research and development, the GMP process has a high probability of causing geometrical defects and glass damage after molding because of the complex thermal–mechanical–chemical behavior which is induced during the molding process at a high temperature and by the rapid temperature change and collective pressure load of the glass [20].

Despite industrial demands, studies on the GMP process are mostly focused on research for optical lenses with aspheric geometry [19,21,22,23,24,25,26] or the implementation of micro-/nano-patterns [25,26,27,28,29,30,31,32,33,34,35,36]. Zhang et al. [6], Kuang et al. [35], and He et al. [36] conducted studies on the GMP process for molding curved glass to achieve a curvature beyond that of the 2.5D cover glass. For a curvature of ≥90°, however, the undercut geometry must be included in the bottom press mold. Nevertheless, research on the design of the press mold of the glass with undercut geometry and the molding process has not yet been reported. Therefore, the technology is limited to the application of the GMP process for manufacturing curved glass with a curvature of <90°.

Herein, to overcome the above-mentioned limitations of previous studies, a mold design plan that can achieve a cover glass with a curvature radius of ≤3 mm and a curvature of ≥90° was proposed; this plan involved a compression process wherein two punches with curved geometry were used during the GMP process in two steps. In addition, the applicability of the mold design plan was verified through analysis and forming experiment. For analyzing the glass properties measured in previous studies and those achieved through the implementation of the mold design plan, the bending geometry of glass subjected to single-step compression forming (hereafter, the single-step process) and two-step compression forming (hereafter, two-step process) was predicted using finite element method (FEM) analysis. A molding product with a curvature radius of 2.5 mm and a curvature of 138.9° was obtained with no damage. This study presents the results of an investigation into the possibility of a two-step local forming method for brittle materials. This study demonstrated that the two-step compression processing is suitable for the local forming of a curvature of more than 90°, as demonstrated experimentally and via simulation, compared with the existing GMP process and single-step compression processing. Although it was not possible to attain full 180° forming in the experimental results, the two-step molding method sufficiently presents a scientific basis for the possibility of locally forming a curvature of more than 90°. This breakthrough should be seen against the background that a curvature of more than 90° was thought to be fundamentally impossible.

## 2. Two-Step Glass Hot Forming Processes

**Figure 1:**
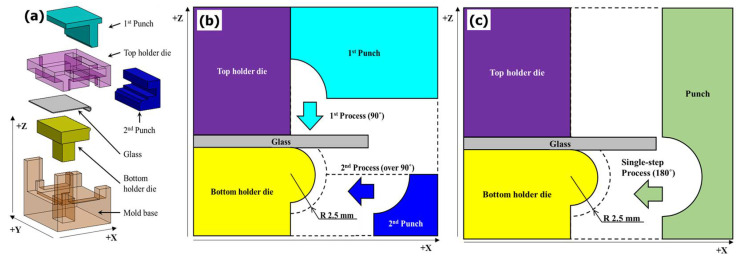
(**a**) GMP design for the molding of the cover glass edge to achieve a curvature of ≥90°. Die and punch geometry for (**b**) 2-step compression forming and (**c**) single-step compression forming.

Two types of processes were considered for the molding of the cover glass edge with a curvature of ≥90°, as shown in Figure 1. In general, the curved geometry of the molds is transferred to the glass after inserting the glass between the top and bottom molds. In this study, however, the GMP mold was designed such that the first punch with the geometry of a concave quadrant (radius: 2.5 mm) can press the glass edge in the –z-direction first to form a curvature of up to 90° and the second punch with the same geometry can press the side of the glass (−x-direction) bended at 90° to form a curvature more than 90°, as shown in Figure 1b. Except for the side of the glass which is expected to form a curvature, the glass is held in place by the top and bottom holder dies. The top holder die has a rectangular hole for the first punch to move in the ±z-direction without any interference. For the bottom holder die, the convex semicircle geometry (radius: 1.7 mm) offset to the quadrant geometry is applied to implement a curvature by pressing the glass positioned between the concave part of the punch and the convex part of the die. First, the possibility of forming the curvature of the glass edge was estimated and compared between the single-step process, which presses the glass from the side using only a semi-circular punch, as shown in Figure 1c, and the two-step process, which uses two punches in sequence, as shown in Figure 1b through molding analysis. Next, the GMP experiment was performed by adopting a process favorable for forming the curvature at the glass edge for the analysis, and the results were compared.

## 3. Analysis of Glass Properties for GMP Analysis

### 3.1. Temperature-Dependent Behavioral Characteristics of Glass

The flow stress (*σ*) of a material subjected to the molding process at high temperature can be expressed as a function of the temperature (*T*), strain (*ε*), and strain rate ($\dot{\varepsilon }$), as in Equation (1) [35]. For glass, Equation (1) can be specified as Equation (2) [37,38].

(1) $$\sigma =\sigma \left(\varepsilon ,\;\dot{\varepsilon },\;T\right)$$

(2) $$\sigma =\eta \left(T\right)\dot{\varepsilon }+E\varepsilon \;$$

In the GMP process, a glass preform was heated to a temperature several tens of degrees Celsius higher than the transition temperature and compressed using the molds located at the top and bottom to replicate the core geometry. The stress distributed in the glass was then reduced by applying a load to the preform over a short period of time at a low cooling rate, and the glass was demolded by rapidly cooling it to room temperature [27,28]. Glass mainly exhibits three types of deformation behavior depending on the temperature condition. In the solidus region at temperatures lower than the transition temperature, the behavior of glass depends only on elastic deformation. In the liquid region, where the temperature is higher than the transition temperature, the deformation of glass depends only on viscosity. In the region of the transition temperature, however, the deformation of glass is determined by temperature-dependent viscoelasticity. Because the glass molding is performed at a high temperature near the softening point (T_s_: the temperature at which the glass moves under its own weight), the glass can serve as an incompressible Newtonian fluid. At temperatures higher than the transition temperature, the effect of initial deformation can be neglected because the behavior of glass does not follow the deformation history. In addition, if the glass molding process is assumed to be an isothermal process and only the compression step is considered among the four steps of heating, compression, annealing, and cooling, Equation (2) can be expressed as Equation (3) [37,38],

(3) $$\sigma =k{\left(\dot{\varepsilon }\right)}^{m},.\;$$

where k is the stress coefficient, m is the strain coefficient, and $\dot{\varepsilon }$ is the strain rate [37,38].

### 3.2. Glass for the GMP Process

**Figure 2:**
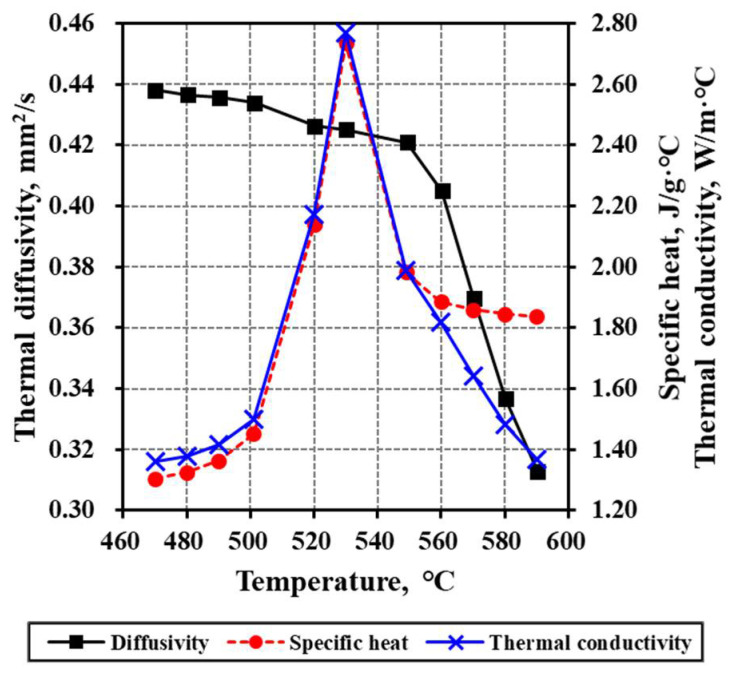
Thermal properties of K-PBK40 according to the temperature.

K-PBK40 (SUMITA Optical Glass Inc., Japan) with an *nd* (Abbe’s number at the 587 mm wavelength) value of 63.5 and a refractive index of 1.517 was selected as the glass to be pressed [38]. The flow stress equations, Equations (4)–(7), were derived on the basis of previous research results (compression test [39]). To briefly describe the compression test, four cylindrical specimens with a diameter of 7 mm and a height of 7 mm were heated to 560 °C or higher. Then, considering that the compression speed (strain rate) was 0.01–0.02 [1/s] during molding in the GMP process, the experimental conditions were determined at the compression test temperatures (560, 570, 580, and 590 °C) and the strain rates (0.005, 0.01, 0.03, and 0.05 1/s). When the temperature measured by the jig pressing the upper and lower surfaces of the glass specimen reached the test temperature, the compression test was performed by compressing the specimen by approximately 60% (4.3 mm) while maintaining the normal state. A stress–true strain curve was derived from the load (N)-displacement (mm) curve for each temperature obtained from the test. Each value of the actual stress for actual strains between 0.1 and 0.8 was converted to the logarithmic value using a temperature-dependent linear curve. Next, a log (true stress)–log(true strain) curve could be obtained by fitting the average value determined by the strain. Finally, Equations (4)–(7) were derived from the log curve and Equation (3). The thermal characteristics of the glass material in the FEM simulation were reflected by also measuring the specific heat, thermal diffusion coefficient, and thermal conductivity, as shown in Figure 2 [40]. The other physical properties provided by the manufacturer are listed in Table 1 [38].

(4) $$\sigma =838.67{\left(\dot{\varepsilon }\right)}^{0.794}\;$$

(5) $$\sigma =427.5{\left(\dot{\varepsilon }\right)}^{0.853}\;$$

(6) $$\sigma =213.3{\left(\dot{\varepsilon }\right)}^{0.893}$$

(7) $$\sigma =104.2{\left(\dot{\varepsilon }\right)}^{0.912}$$

## 4. Finite Elemental Analysis of Glass Molding Press

### 4.1. Analysis Geometry and Condition

**Figure 3:**
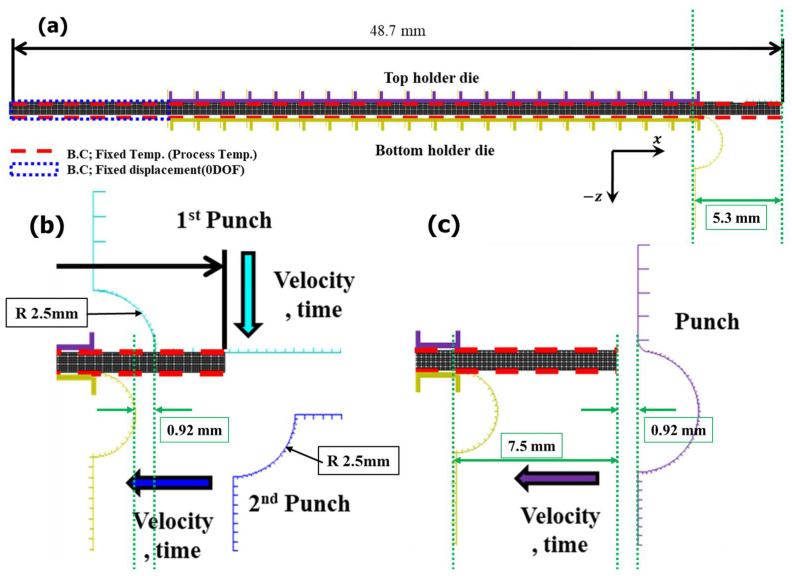
Geometry for GMP process simulation: (**a**) overall geometry, (**b**) fixed/loaded condition of 2-step process, and (**c**) fixed/loaded condition of single-step process.

As mentioned in Section 3.1, a glass edge can be molded to achieve a curvature of more than 90° by the single-step and two-step processes. Computer-aided engineering (CAE) analysis was conducted to simulate the deformation behavior of the glass in the molding process as a function of the punch speed and forming temperature. The simulation was performed using MARC (2005), commercial nonlinear finite element model (FEM) software. The 2D model shown in Figure 3 was built for the analysis, and the radius of each punch was set to 2.5 mm. To avoid unnecessary stress concentrations, a fillet radius of 0.2 mm was placed on the edge of the punch that was in contact with the glass.

An FEM model of the glass with a thickness of 0.8 mm was constructed by applying a mesh of quadratic four-node surface elements. The numbers of nodes and elements were 4392 and 3896, respectively. The die and punch were assumed to be rigid bodies. In a previous simulation, a cylindrical specimen of the same glass material with a diameter of 7 mm and a height of 7 mm was compressed by 4.2 mm in the direction of gravity at 570 °C. The results of the analysis, in which the friction coefficient was varied in increments of 0.1 from 0 to 1, showed that the model most similar to the actual compression test result was that of which the friction coefficient was set to 1 [41]. The forming temperature and punch speed were the independent variables for the process simulation. The detailed analysis conditions are presented in Table 2. Furthermore, this study established an experimental plan, with the detailed goal of deriving the minimum forming temperature and maximum jig feed speed that can shorten the total forming process time while implementing a 180° angle of curvature. The punch speed and forming temperature (process temperature) were selected as variables, because even for the same glass material, the strain–stress curve graph changes according to the strain rate in the same temperature environment, and the strain–stress curve graph varies according to temperature even though the same strain rate is applied [41].

### 4.2. Analysis Results

#### 4.2.1. Single-Step Compression Forming (Simultaneous Forming Up to 180°)

**Figure 4:**
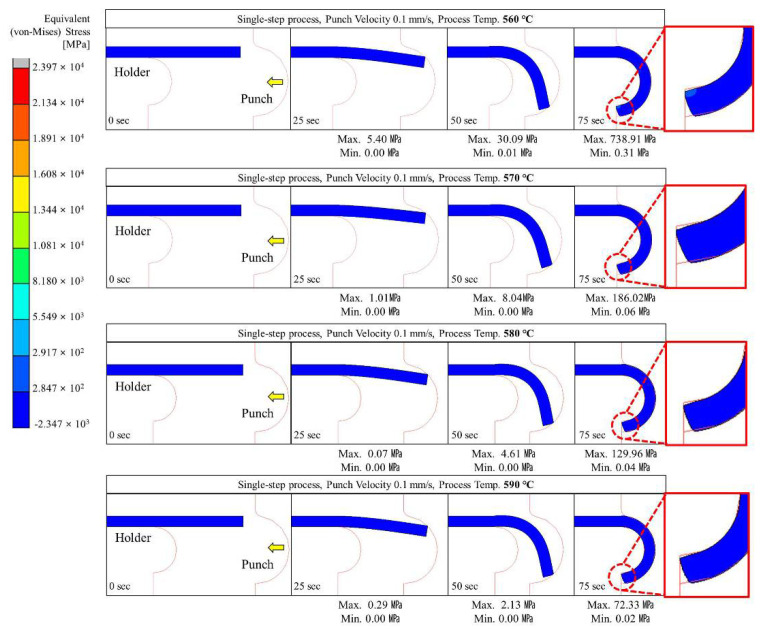
Stress distribution on glass during the single-step process with a punch velocity of 0.1 mm/s.

**Figure 5:**
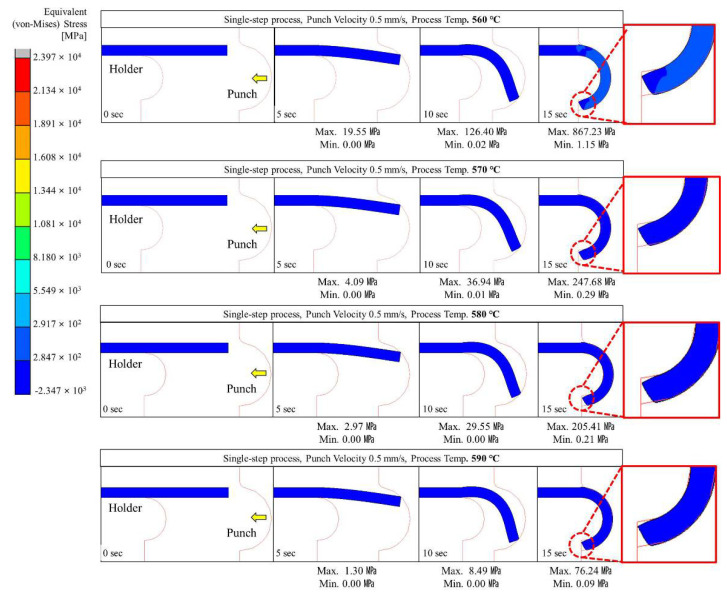
Stress distribution on glass during the single-step process at a punch velocity of 0.5 mm/s.

**Figure 6:**
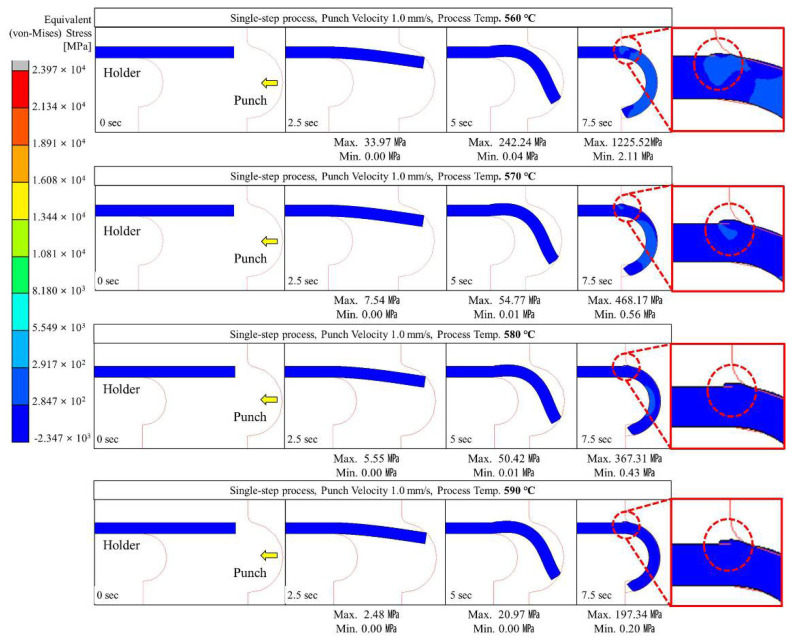
Stress distribution on glass during single-step compression forming for a punch speed of 1 mm/s.

**Figure 7:**
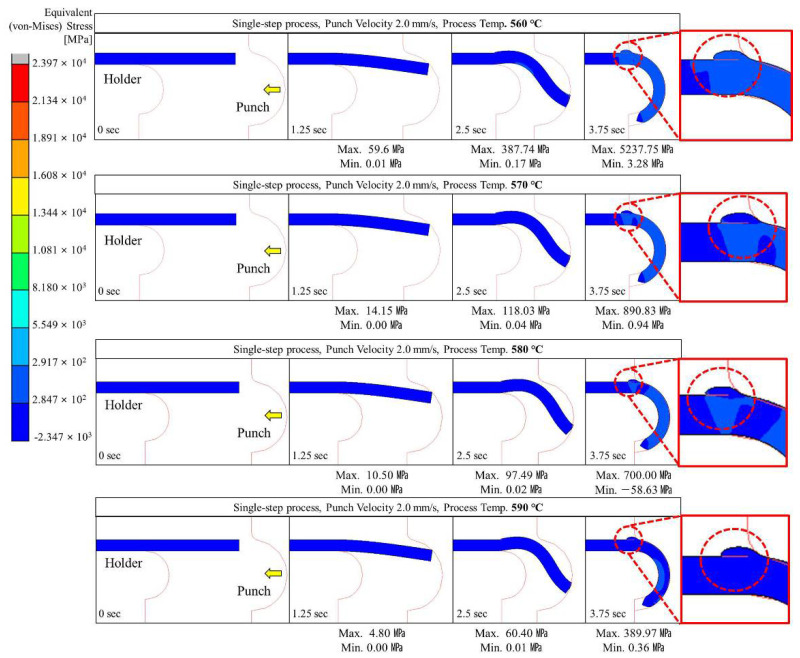
Stress distribution on glass during single-step compression forming at a punch speed of 2 mm/s.

**Figure 8:**
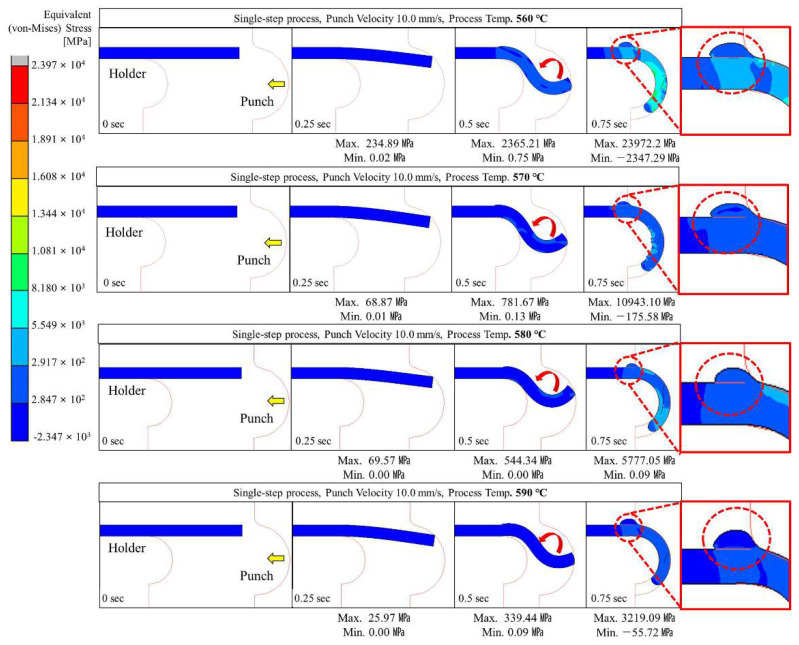
Stress distribution on glass during single-step compression forming at a punch speed of 10 mm/s.

Glass hot forming in which the semi-circular punch was used was analyzed to examine the possibility of manufacturing glass with a curvature of 180° using the single-step process; the results are shown in Figure 4, Figure 5, Figure 6, Figure 7 and Figure 8. It was found that the cavity in the closed mold was not sufficiently filled regardless of the punch speed and processing temperature. As shown in Figure 5, at a forming temperature of 560 °C and a punch speed of 0.5 mm/s, the glass edge was subjected to stress of 867.23 MPa, which is higher than the fracture strength of the material. This indicates that local fracture may occur when the glass is assembled with other parts in the post-processing stage.

As shown in Figure 4, Figure 5, Figure 6, Figure 7 and Figure 8, the maximum stress applied to the glass increased regardless of the forming temperature, as the punch speed increased from 0.1 to 10 mm/s. It was observed that the stress acting on the glass was distributed over the curved section, including the edge end and the area in contact with the punch edge. When the punch speed was 1 mm/s or higher, the mold was gradually closed and the stress was applied in the opposite direction to the induced geometry due to an increase in the area in contact with the punch. As a result, as indicated by the red dotted circles in Figure 6, Figure 7 and Figure 8, the material was crushed at the top of the punch. In other words, in the single-step process, the cavity was filled insufficiently with glass, and surface irregularities occurred in the glass making contact with the top surface of the punch, indicating that the process is not suitable for glass molding with a curvature of 90° or more. Even an attempt to improve the fluidity of the glass material by increasing the forming temperature from 560 to 570, 580, and 590 °C resulted in the same phenomenon of irregularities on the upper surface of the glass material in the single-step process when the punch speed was 1 mm/s or more. In addition, at a forming temperature of 560 °C, the maximum stress higher than the fracture strength of the glass was observed for a punch speed of 0.5 mm/s or higher. Therefore, the molding temperature of 560 °C in the single-step compression process seems to have the narrowest punch speed window.

#### 4.2.2. Two-Step Compression Forming (Sequential Forming to 90° and 180°)

**Figure 9:**
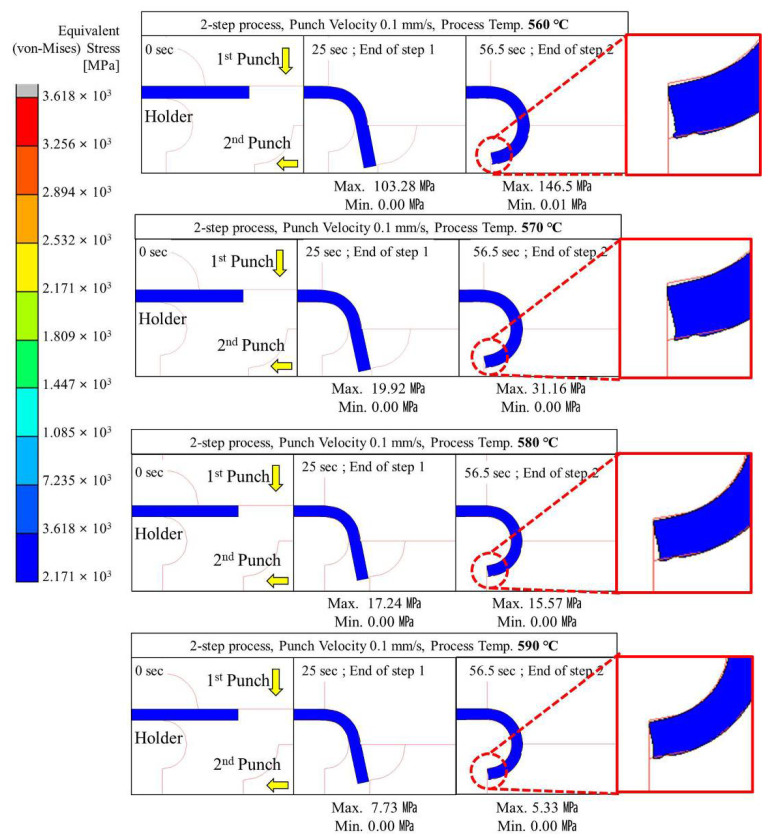
Stress distribution on glass during 2-step compression forming at a punch speed of 0.1 mm/s.

**Figure 10:**
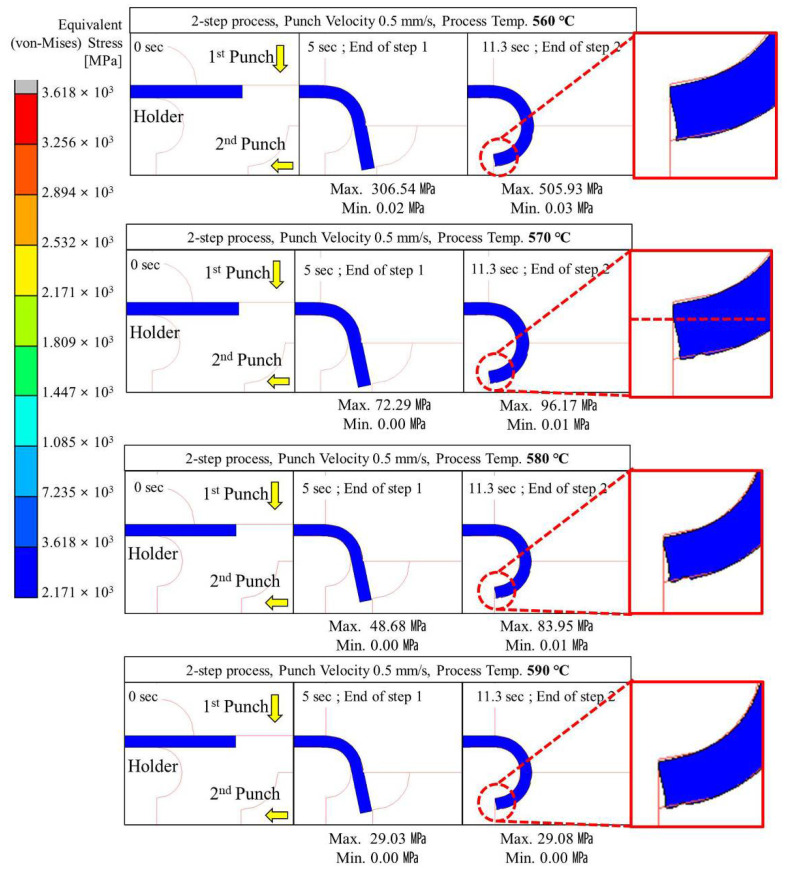
Stress distribution on glass during 2-step compression forming at a punch speed of 0.5 mm/s.

**Figure 11:**
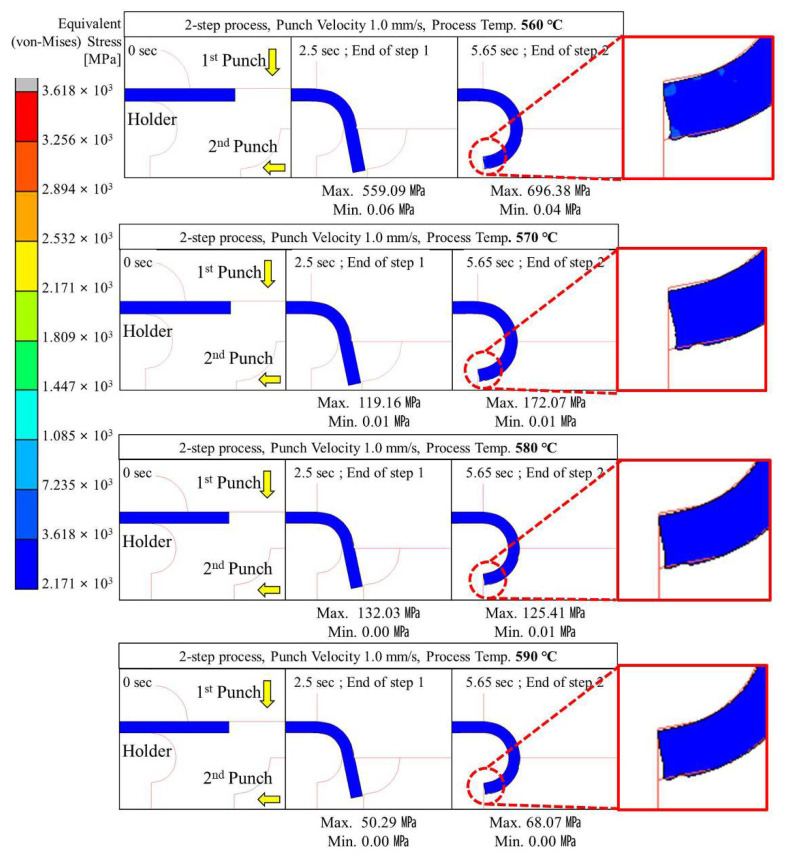
Stress distribution on glass during 2-step compression forming at a punch speed of 1 mm/s.

**Figure 12:**
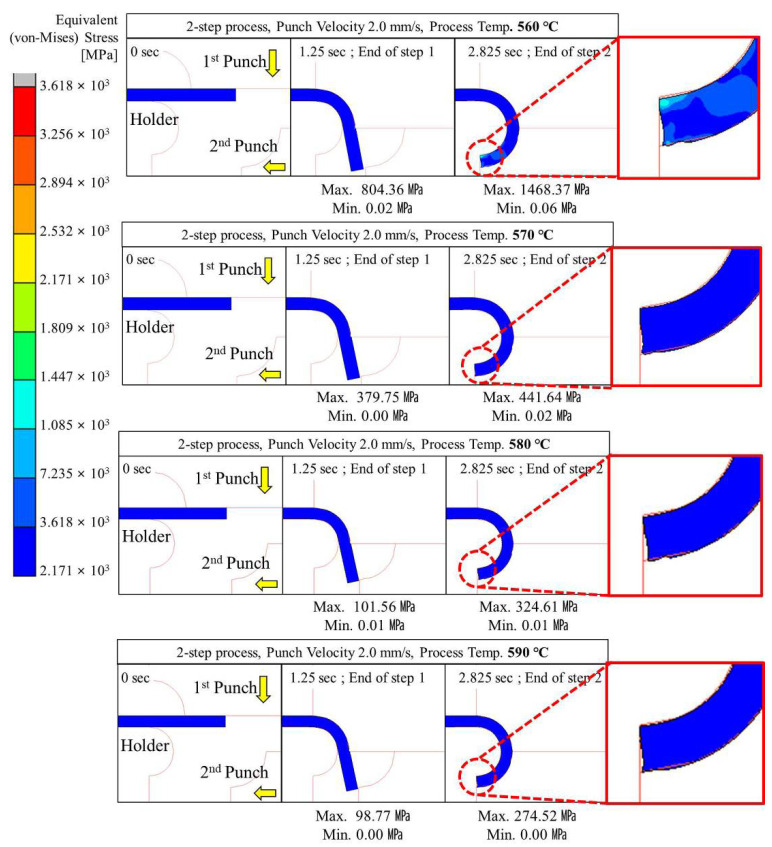
Stress distribution on glass during 2-step compression forming at a punch speed of 2 mm/s.

**Figure 13:**
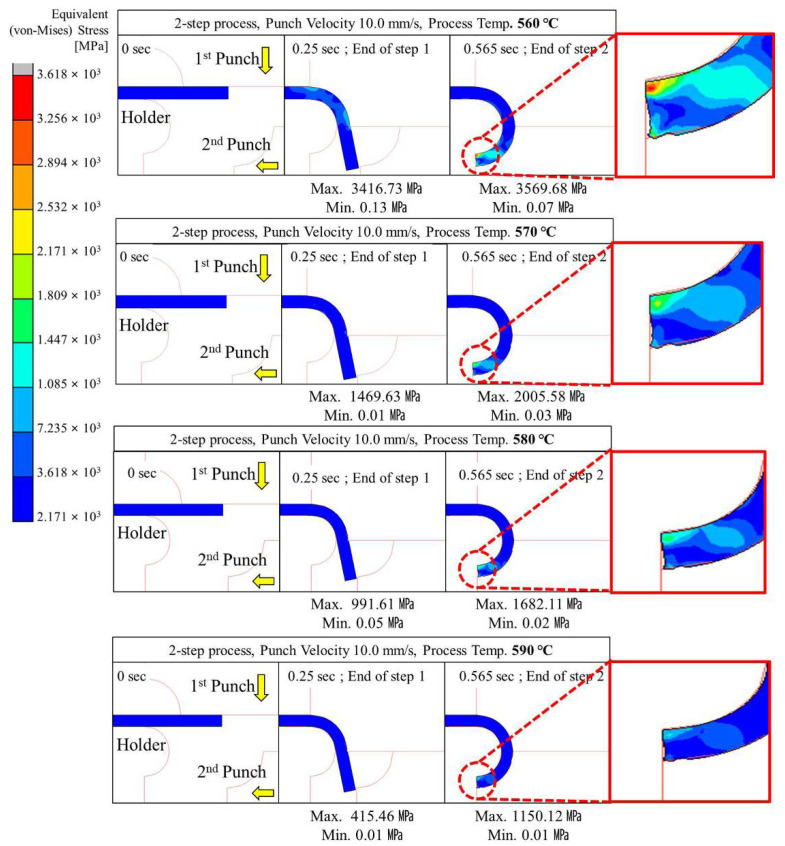
Stress distribution on glass during 2-step compression forming at a punch speed of 10 mm/s.

Two-step glass hot forming with two quadrant-shaped punches in the sequence was analyzed to examine the possibility of manufacturing glass with a curvature of more than 90° using the two-step process; the results are shown in Figure 9, Figure 10, Figure 11, Figure 12 and Figure 13. For the same forming temperature and punch speed as that of the single-step process, the stress generated on the glass in the two-step process decreased to 58, which is 14.9% of that acting on the glass in the single-step process. In addition, the cavity in the closed mold was much more optimally filled than the single-step process, and no surface irregularity occurred in the area of the glass in contact with the punch edge. Two-step compression molding analysis applying the forming temperatures of 580 and 590 °C found that the glass material was not fractured at the feed rate of 2 mm/s was the case with the forming temperature of 570 °C. The free lengths of the glass material used in both processes were the same (10 × 40 mm). To implement the 180° angle of curvature specified as the research goal, the radius of curvature after thermoforming should be 2.5 mm, and the length of the glass from which the curvature is formed should be 15.7 mm. Therefore, considering that the length of the glass increases with temperature and compression, the die and jig were designed to achieve a length of 5.3 mm for the glass material not fixed by the top and bottom holder dies (page 7 line 189; see Figure 3). In step compression molding and two-step compression molding, the distance at the first contact between the glass material protruding from the die and the punch is the same at 0.92 mm. For a glass material (K-PBK 40) with a Young’s modulus (E) of 799 GPa at room temperature, the value of E decreases to 1/10 or less when it falls within the forming temperature range. Assuming that the value of E in the forming temperature range is 80 GPa, single-step compression molding is a process in which the left side is fixed and a load is applied in the −x-axis direction. Therefore, the critical load (buckling critical force/stress) (*P_cr_*) can be calculated using Equation (8), as suggested by Euler [42]. Thus, the buckling critical force/stress (*P_cr_*) can be calculated by the Euler formula as expressed in Equation (8) [42].

(8) $$P_{cr}=\frac{4\pi^{2}EI}{L^{2}}$$

The critical load calculated using the above equation for the 10 × 40 × 0.8 mm glass material used in this study was 997.12 GPa. This suggests that the deformation in the glass specimen during the single-step compression process is not due to the buckling effect. Moreover, to prevent the buckling effect, multiple molds with different curvatures should be sequentially provided, the distance between the glass material and the upper and lower molds should be minimized, and multi-stage molding such as the GPM continuous process or progressive molding is required. Hence, this process can be more complicated than the single-step or two-step compression molding mentioned in this study.

The maximum stress distributed in the glass was higher than the fracture strength of the glass at a punch speed of 2 mm/s or higher at a forming temperature of 560 °C and at a punch speed of 10 mm/s or higher at a forming temperature of 570 °C. The above analysis results indicate that the two-step GMP is suitable for curving the glass edge with a curvature of 90° or more without surface irregularity. Thus, the optimal conditions for the two-step process are a punch speed between 0.1 and 1.0 mm/s at 560 °C or 570 °C.

## 5. Experiments

### 5.1. Equipment

**Figure 14:**
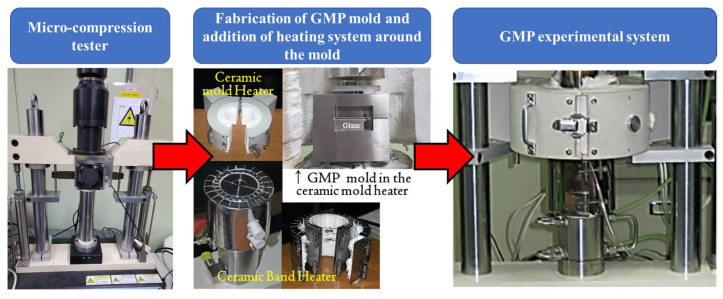
GMP experimental system for 2-step glass molding process.

Figure 14 shows the molding equipment that was developed for the experimental verification of the two-step process. The ceramic mold heater was used to directly heat the test mold, and its maximum temperature was 700 °C. The set-point temperature was adjustable in steps of ±0.2 °C using the temperature controller (UP550, Yokogawa Electric Corporation, Musashino, Japan). In addition, two probe-type displacement sensors (Soltron Corporation, Suwon-si, South Korea, Peak to Peak error 0.99 μm, 10 mm reading) were installed to control the molding position with their correction values. The experimental system also included a load cell capable of measuring loads up to 2200 N with an error range of ±0.5%. The maximum test speed was 1 mm/sec with a displacement accuracy of ±0.01% at a maximum stroke of 10 mm [43].

### 5.2. Experimental Setup

**Figure 15:**
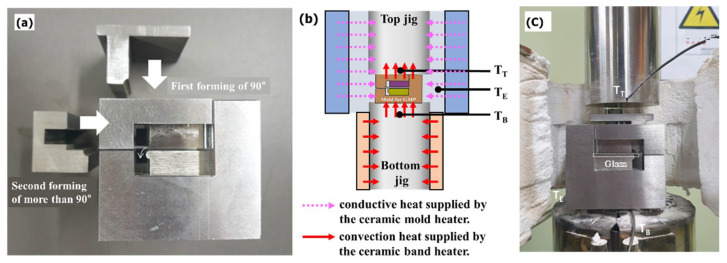
(**a**) Fabricated mold/punch (**b**) schematic of the mold heating system (T_T_ (temperature of top jig, T_E_ (temperature of exterior of mold), and T_B_ (temperature of bottom jig)) and (**c**) assembly of heating system and mold/punch.

All components used for GMP molding, including the mold, punches, and holders, were produced from STD61, as shown in Figure 9. As shown in Figure 15, the mold was heated from room temperature to the molding temperature using the ceramic mold heater, after which the molding temperature was maintained by heating using the ceramic band heater.

The glass we used in the experiment was K-PBK40 (SUMITA Optical Glass Inc.). As shown in Figure 1b and Figure 15, the two-step process was performed by fixing the glass between the holder dies at the top and bottom. The glass was bent by 90° by the quadrant-shaped first punch, which approached the glass in the vertical direction, and then the quadrant-shaped second punch closed and pushed in the lateral direction such that the glass edge could be rolled with a curvature of more than 90° upon the completion of molding. Here, a punch speed of 1 mm/s, which is the fastest feed speed, and a forming temperature of 570 °C were applied. Details of the process parameters are presented in Table 3. These results were used to calculate the stress of the breaking strength, for example 867.23 × 0.25 = 216 MPa. A safety factor of 4 was considered when designing for glass materials.

### 5.3. Experimental Results

**Figure 16:**
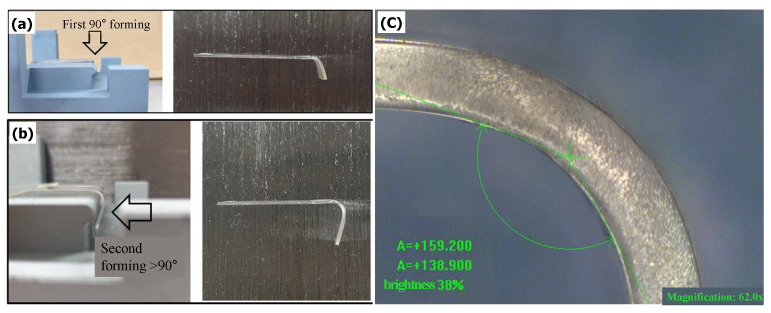
(**a**) First-forming results, (**b**) second-forming results, and (**c**) magnification of the formed glass curvature.

A molding product with a curvature radius of 2.5 mm and an edge curvature of 138.9°, which differs from that in the simulation, was obtained, as shown in Figure 16. This discrepancy could be explained as follows. First, of all the steps in the GMP process (heating, compression, annealing, and cooling), the analysis only considered the compression step, i.e., the flow stress of the glass in the compression step, the stress relaxation in the compression step, the structural relaxation in the annealing and cooling steps, and the heat transfer/heat exchange phenomena applied to the mold and glass in the compression → annealing → cooling steps were not considered. Second, the annealing and rapid cooling steps were omitted from the actual hot forming experiment, unlike in previous studies. This appears to have caused a type of spring-back phenomenon which is known to occur when a glass-molding product with an edge curvature of 180° is released from the GMP mold while cooling to room temperature without removing the residual stress [43]. In the future, further research on GMP simulation and molding, which takes into account the annealing and cooling steps, is planned.

To investigate the effect of the two-step molding process on the thickness, the thickness of the bent area was measured using a microscope and image-processing tool, as shown in Figure 16c. Unlike the other GMP processes or injection molding, the device (Figure 15) and molds (Figure 1 and Figure 16) used in this study were produced by open local molding where the glass material was not fully sealed in the mold, which is different from general sealed molding. Thus, the change in the volume could be indirectly evaluated by measuring the shape (e.g., the thickness and xy-profile) of the glass material after the compression step was completed, instead of conducting a general volume assessment. The thickness variations at three measuring points are listed in Table 4. In addition, the variations in the surface roughness before and after forming are compared in Table 5. The 10-point average roughness (Rz) value of 30.211 μm was similar to the average thickness of 32.48 μm in the analysis results; therefore, the simulation predictions are in good agreement with the experimental results.

## 6. Conclusions

In this study, a mold design plan that can achieve a curvature radius of 3 mm or less and a curvature of 90° or more was proposed by dividing the existing GMP process into two steps. Then, the mold design plan was verified by conducting simulation analysis and forming experiments. Based on the results, the following conclusions are drawn:

(1) The FEM simulation results showed that it is possible to form a glass edge with a curvature of 180° without damaging the material and without introducing surface irregularities by applying two-step compression forming with quadrant-shaped (curvature 90° + 90°) punches rather than single-step compression forming with a semi-circular (curvature 180°) punch during the GMP process for curved glass molding.
(2) The optimal conditions for two-step compression forming to form a curved edge with a curvature of 90° or more using glass of 10 mm (width) × 40 mm (length) × 0.8 mm (thickness) were derived using plastic analysis and are as follows: punch speeds of less than 2 mm/s and 10 mm/s at forming temperatures of 560 °C and 570 °C, respectively.
(3) An actual glass forming experiment performed by applying a punch speed of 1 mm/s at a forming temperature of 570 °C resulted in formed glass with a curvature radius of 2.5 mm and a thickness deviation of 30 μm, similar to the analysis results. The curvature, however, was found to be 138.9° instead of 180°. This may be the result of the overestimation of the curvature in the analysis and the spring-back phenomenon occurred in the actual test specimen due to the unremoved residual stress, because the annealing and cooling steps were not properly implemented in the FEM simulation and forming experiments.
(4) This study made it possible to form a curvature edge of more than 90° by only thermoforming a part of the upper and lower mold shapes used to perform the GMP process. This approach obviated the need to apply heat pressing to the entire upper and lower surfaces of the cover glass.
(5) The mold design method that is mainly used in the conventional GMP process cannot apply the undercut structure for molding and extracting the edge surface with a curvature of more than 90°. This study overcame this problem by proposing a mold that can mold cover glass with an edge surface of 90° or more, even if the shape of the top and bottom of the mold does not correspond to the curvature shape of the cover glass to be created.

## 7. Discussion

This study explored the possibility of a two-step local forming method for brittle materials. The experimental and simulation results suggested that the two-step compression processing applied in this study is suitable for the local molding of an angle of curvature of more than 90° compared with the existing GMP and single-step compression processes. Although it was not possible to perform a full 180° forming as shown in the interpretation of the experimental results, this study presented the scientific basis for the possibility of local forming of more than 90° with the two-step forming method. This constitutes a breakthrough in that forming in excess of 90° was considered fundamentally impossible. In the future, technologies for examining the GMP mold design and molding process that can form curved glass with a single curvature of 90° or more and multiple curvatures in advance are expected to be developed. This would require further research using GMP simulation and molding that takes into account the annealing and cooling processes. This study did not use multiple molds and thermal heating/cooling devices in each process, as in the general GMP process [39]. Instead, a single device was used for heating and subsequent cooling. Moreover, the equipment driving variable was displacement control rather than load control. Thus, when the volume of the thermoformed glass specimen shrinks in the mold during the annealing and rapid cooling process, a load could not be applied to prevent the glass from returning to its original shape (spring-back phenomenon). In the future, we plan to conduct research on optimizing the load conditions for volume shrinkage compensation during the annealing and rapid cooling processes. This would involve upgrading the equipment to enable load control in addition to displacement control during mold and jig feed even if the devices are not the same as for the general GMP process.

## Figures and Tables

**Table 1:** Material properties of glass (K-PBK40).

Category	Properties	Unit	Value
Thermal	Transformation Point	T_g_ (°C)	501
	Yielding Point	A_t_ (°C)	549
	Thermal Expansion	100–300 °C (10^−6^/°C)	7.3
Mechanical	Young’s Modulus	E (GPa)	799
	Modulus of Rigidity	G (GPa)	325
	Poisson’s Ratio	σ	0.229
Other	Specific Gravity	s.g	2.39

**Table 2:** Analysis conditions of glass press molding.

Analysis Condition		
Condition	Single-Step Process	2-Step Process
Initial/process temperature on glass and die and punch, °C	560/570/580/590 °C	
Boundary condition	Fixed temperature: red dashed lines/glass top and bottom/process temperature (560, 570, 580, 590 °C) Fixed displacement: nodes in blue dot box/0 DOF	
Mold moving speed (mm/s) 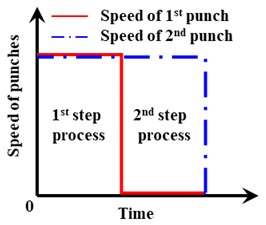	Punch: −x-direction/0.1, 0.5, 1, 2, 10 mm/s	1st Punch: −x-direction/0.1, 0.5, 1, 2, 10 mm/s 2nd Punch: y-direction/same speed as 1st Punch
K	838.67 (@560 °C)/427.4 (@570 °C)/213.3 (@580 °C)/104.2 (@590 °C)	
M	0.794 (@560 °C)/0.853 (@570 °C)/0.893 (@580 °C)/0.912 (@580 °C)	
Coefficient of friction	1	

**Table 3:** Indication of the maximum stress value applied to the glass material by each molding process ^1^.

Temperature (°C)/Punch Speed (mm/s)	Single-Step Compression Process					2-Step, Compression Process				
	0.1	0.5	1	2	10	0.1	0.5	1	2	10
560	738.91	867.23	1225.52	5237.75	23,972.2	146.50	505.93	616.38	1468.37	3569.68
570	186.02	247.68	468.17	9808.83	10,943.1	31.16	96.17	172.07 ^1^	441.64	2005.58
580	129.96	205.41	367.31	700.00	5777.05	15.57	83.95	125.41	324.61	1682.11
590	72.33	76.24	197.34	389.97	3219.07	5.33	29.08	68.07	274.52	1150.12

^1^ Blue text: process conditions applied to the experiment; red cells: non-formable section derived above the maximum flexural strength of the material.

**Table 4:** Thickness observation after glass molding analysis.

Measuring Position		2-Step Process (Dimension: mm)							
		570 °C				560 °C			
		Pt.1	Pt.2	Pt.3	Avg. (L-L0)	Pt.1	Pt.2	Pt.3	Avg. (L-L0)
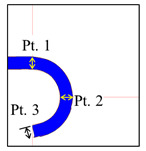	0.1	0.771	0.767	0.758	0.035	0.768	0.765	0.758	0.036
	0.5	0.770	0.768	0.764	0.033	0.772	0.765	0.763	0.033
	1	0.773	0.768	0.762	0.032	0.771	0.765	0.767	0.032
	2	0.769	0.765	0.785	0.027	0.769	0.766	0.763	0.034
	10	0.774	0.766	0.782	0.026	0.773	0.764	0.775	0.029

**Table 5:** Surface roughness observation after 2-step glass molding.

Measuring Position	(Dimension: μm)	Ra	Rz
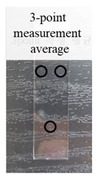	Before forming	0.31	1.88
	After 2-step process	2.435	30.211

## Data Availability

Not applicable.
